# Posterior Cortical Atrophy: Altered Language Processing System Connectivity and Its Implications on Language Comprehension and Production

**DOI:** 10.3390/brainsci15121287

**Published:** 2025-11-29

**Authors:** Neha Singh-Reilly, Ryota Satoh, Hiroyuki Watanabe, Jonathan Graff-Radford, Mary M. Machulda, Val J. Lowe, Keith A. Josephs, Jennifer L. Whitwell

**Affiliations:** 1 Department of Radiology, Mayo Clinic, 200 1st St SW, Rochester, MN 55905, USA; 2 Department of Neurology, Mayo Clinic, 200 1st St SW, Rochester, MN 55905, USA; 3 Department of Psychiatry & Psychology, Mayo Clinic, 200 1st St SW, Rochester, MN 55905, USA

**Keywords:** posterior cortical atrophy, language processing system, gray matter volume loss, clinical performance, resting state fMRI

## Abstract

**Background**: Posterior cortical atrophy (PCA) patients typically show progressive visual decline, although language impairment can be a prominent feature. Previous studies have explored language connectivity impairment in PCA; not much is known about the exact nature of language processing disruptions in PCA. **Methods**: Thirty-seven PCA patients were compared with 39 cognitively unimpaired (CU) individuals. Functional connectivity was evaluated for the language processing system, which consisted of language, perceptual, motor, and cognitive networks. Linear regression was performed to compare within- and between-network connectivity across networks and within the language network in PCA compared to CU individuals. Relationships between language network connectivity, clinical performance, and gray matter volumes were also evaluated. **Results**: PCA showed reduced connectivity within the language and perceptual networks, along with reduced between-network connectivity compared to CU individuals. Regional connectivity breakdown within the language network was also noted. Language processing system connectivity was not associated with clinical performance on language processing tests. However, a breakdown in Broca-Wernicke, Wernicke-middle frontal gyrus, and Wernicke-frontal orbital gyrus connectivity was related to worse performance on sentence repetition. Lastly, gray matter volume was not associated with language network connectivity. **Discussion**: Together, these results reiterate the breakdown of language network connectivity in PCA and highlight the disruption in connectivity at the regional level within the language network. Reduced connectivity within the perceptual network and associations between reduced language network connectivity and worse performance on sentence repetition imply a breakdown within the language processing systems in PCA.

## 1. Introduction

### 1.1. Background

Posterior cortical atrophy (PCA) is characterized by progressive decline in visual functions and is often referred to as the visual variant of Alzheimer’s disease (AD) [1]. Patients with PCA have difficulties navigating their surroundings and perceiving objects [2] as well as simultanagnosia, optic ataxia (Balint syndrome), and oculomotor apraxia [1]. From a neuroimaging standpoint, PCA patients show parieto-occipital atrophy, hypometabolism, and tau deposition [3,4,5], along with reduced functional connectivity in the visual network [6].

Besides these prominent visual characteristics, several studies with PCA have described the presence of language impairment. More specifically, patients with PCA can have progressive language deficits [7], with impaired reading, writing [7,8], fluency [7], syntactic comprehension [9], anomia, and slowed speech rate [9,10,11]. Deficits in sentence repetition [9], auditory input processing [9], and the presence of phonological errors [12] have also been noted in PCA. Additionally, left temporal lobe atrophy and tau deposition have been noted to occur [13], with reduced functional connectivity in the language network [6,14]. Together, these findings suggest that there is much to be understood about the nature of language dysfunction in PCA.

Previous studies have explored language dysfunction at the clinical [13,15,16] and neuroimaging levels through exploration of associations between imaging modalities and clinical testing [17] or at the functional level through exploration of connectivity disruptions in the classical language network, which includes Broca’s and Wernicke’s areas [6,14]. However, areas for language processing extend beyond these classical hubs, as evidenced by a recent study that leveraged functional connectivity data from 806 neurotypical adults (i.e., adults with no history of neurological and language impairments) to identify several brain areas that are specialized for language. These regions included the inferior and middle frontal gyri, the frontal orbital gyrus, the middle and superior temporal gyri, and the temporal pole [18]. Although language processing is the core function of these brain areas, it is a sophisticated communication system that comprises two distinct operations: language comprehension and production.

During language comprehension, linguistic signals are perceived through the perceptual network and decoded by the language network [19,20,21]. In contrast, during language production, linguistic messages are encoded by the language network and delivered to the motor network to produce a physical output [19,22]. Hence, a vital component of the language processing system is the language network, as it is involved in both comprehension of linguistic input [23,24] and the generation of linguistic content [23,25]. However, in real-life communication, perceptual and motor networks also play an important role in language processing. They are intricately linked and activated simultaneously during language use (e.g., to listen and produce spoken language to effectively carry out a conversation), despite being distinct networks [26,27] that are not language-selective (i.e., not sensitive to the meaning of linguistic messages) [28,29]. Another important network involved in language processing is the cognitive network that updates existing knowledge structures during language comprehension and supports the ability to think and formulate linguistic messages during language production [30,31,32]. Thus, the cognitive network supports thought, knowledge, and reasoning and works in combination with the language network to support language use in the real world.

### 1.2. Aims of the Study

The main aim of this study was to investigate connectivity alterations within the language processing system. First, we aimed to interrogate within-network functional connectivity of the four networks, i.e., language, perceptual, motor, and cognitive networks, the association between language and other network connectivity, and regional connectivity within the language network in PCA compared to CU individuals. We hypothesized a reduction in within-network connectivity of the language and cognitive networks, with reduced between-network connectivity between these networks. This theory is based on previous studies showing reduced within-network connectivity in the classical language network (consisting of Broca and Wernicke areas only) [6,14] and impaired cognition in PCA patients [33]. We also hypothesized reduced connectivity between the Broca and Wernicke areas in PCA based on previous findings [6,14]. The second aim was to investigate whether the health of the language processing system was related to language performance on clinical testing. More specifically, we looked at the relationship of (i) language comprehension tests with language network within-network connectivity, language-perceptual and language-cognitive connectivity; (ii) language production tests with language network within-network connectivity, language-motor and language-cognitive connectivity; and (iii) language processing tests with regional connectivity within the language network. We hypothesized the absence of a relationship or presence of a weak relationship between comprehension, production, and functional connectivity based on previous findings showing relatively preserved language comprehension and production skills in PCA [9]. The third aim was to determine whether the health of the language network was related to volume loss in brain areas associated with language functions. We did not expect to see a relationship between functional connectivity and structural atrophy because functional connectivity changes precede measurable structural atrophy [34].

## 2. Materials and Methods

### 2.1. Patients

Thirty-seven Aβ-positive PCA patients who fulfilled clinical criteria [1] and 39 Aβ-negative cognitively unimpaired (CU) individuals were recruited by the Neurodegenerative Research Group (NRG) from the Department of Neurology, Mayo Clinic, Rochester, MN, between 16 December 2019 and 1 January 2025. Only participants who were scanned on a 3T volumetric MRI with Siemens scanners were included in this study. Patients diagnosed with primary progressive aphasia, specifically the logopenic variant, were excluded from the study. All patients underwent neurological evaluations (KAJ or JGR), neuropsychological testing (MMM), and a structural MRI with a resting-state functional MRI (rsfMRI) protocol. Pittsburgh Compound B (PiB) positron emission tomography (PET) was performed to investigate evidence of Aβ deposition based on established cut-offs for Aβ positivity [35].

### 2.2. Clinical Testing

Clinical evaluations assessed general cognition using the Montreal Cognitive Assessment (MoCA) [36] and simultanagnosia using a battery of tests that include the Ishihara color plates, images of overlapping line drawings, color images of complex picture scenes, and Navon figures [6]. Visuospatial and visuoperceptual ability were assessed using the Cube Analysis and Incomplete Letters from the Visual Object and Space Perception Battery (VOSP) [37]. Assessment of language comprehension included evaluation of word knowledge using the word-word version of the Pyramids and Palm Trees (PPT) [38] and sentence repetition using the Boston Diagnostic Aphasia Exam (BDAE) repetition subtest [39]. Assessment of language production included evaluation of confrontational naming using the 15-item Boston Naming Test—short form (BNT-SF) [40], lexical and category fluency performance using the letter and animal fluency tests, respectively [41], and the BDAE repetition subset. The validity and reliability scores for these clinical tests are included in Supplementary Table S1.

### 2.3. Image Acquisition

Participants were scanned using a 3T volumetric MRI with Siemens scanners (Magnetom Prisma, Siemens Healthineers (Erlangen, Germany)) at Mayo Clinic, Rochester, MN. The scan included a 3D magnetization-prepared rapid gradient echo (MPRAGE) sequence and a gradient echo planar imaging (TE = 30 ms; slice thickness = 3.3 mm, in-plane resolution = 3.3 mm, and 160 volumes) for rsfMRI scanning [42,43]. Participants were instructed to keep their eyes open during the scan. All scans met fMRI protocol standards, motion parameters, and quality control measures.

### 2.4. Image Processing

The CONN functional connectivity toolbox was used to preprocess all anatomical and functional scans [44] (www.nitrc.org/projects/conn, accessed on 31 March 2025). Preprocessing included discarding the first ten volumes to generate a steady-state magnetization. The first scan was used as a reference image, and all scans were coregistered to this scan. Next steps included slice time correction, realignment (motion estimation and correction) with outlier detection, segmentation, direct normalization to MNI template space, smoothing with a Gaussian kernel of 6 mm full width at half maximum, nuisance regression for white matter, cerebrospinal fluid (CSF) signal, denoising for the first and second order derivatives of the six head motion parameters [45], and bandpass filtering in the 0.01–0.1 Hz frequency to reduce low-frequency drift and noise effects [46]. After preprocessing, all functional images were parcellated using a modified Harvard-Oxford atlas, the CONN network parcellation atlas, and the multiple demand network (MDN) and theory of mind (ToM) atlases [18]. The mean blood-oxygen-level-dependent (BOLD) time series within each region of interest (ROI) of the atlas was extracted, and Pearson’s correlation coefficients were calculated across all ROIs. These values were transformed into Fisher’s R-to-Z transformations.

**Figure 1:**
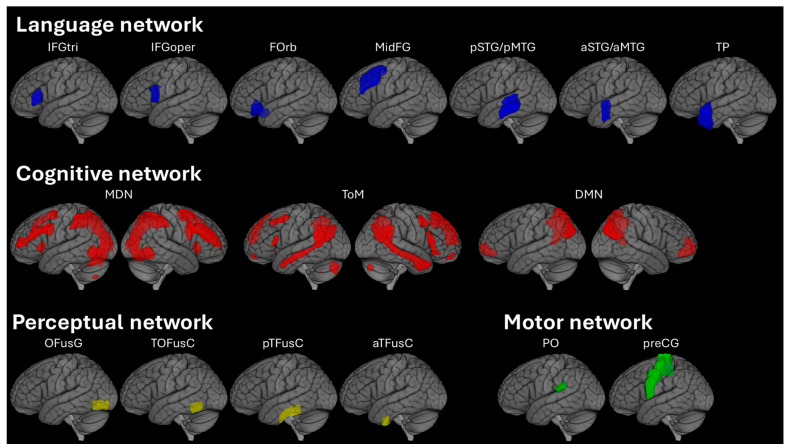
**Network visualization.** Each row includes the location of the regions of interest (ROIs) within each network. The language (blue), perceptual (yellow), and motor (green) networks had ROIs only on the left hemisphere, while the cognitive (red) network was bilateral. Regions greater than 0.3 were shown in the MDN and ToM atlases. This rendered figure was created using the MRIcroGL [60]. Key: IFGtri, inferior frontal gyrus pars triangularis; IFGoper, inferior frontal gyrus pars operculum; FOrb, inferior frontal orbital gyrus; MidFG, middle frontal gyrus; pSTG, posterior superior temporal gyrus aka Wernicke’s area; pMTG, posterior middle temporal gyrus; aSTG, anterior superior temporal gyrus; aMTG, anterior middle temporal gyrus; TP, temporal pole; MDN, multiple demand network; ToM, theory of mind; DMN, default mode network; OFusG, occipital fusiform gyrus; TOFusC, temporo-occipital fusiform cortex; pTFusC, posterior temporal fusiform cortex; aTFusC, anterior temporal fusiform cortex; PO, parietal operculum; preCG, precentral gyrus.

Functional connectivity was evaluated for four networks, namely, (i) the language network, (ii) the cognitive network, (iii) the perceptual network, and (iv) the motor network, using the above-mentioned atlases. The language network was defined using specific ROIs from the Harvard-Oxford atlas, which included anatomical correlates of the functional language network (Lan A) generated using fMRI data from >800 individuals [18]. All the ROIs in this network were on the left hemisphere and included the inferior frontal gyrus (including the pars triangularis and operculum) (Broca’s area), inferior frontal orbital gyrus (FOrb), middle frontal gyrus (midFG), posterior middle temporal gyrus (pMTG), anterior middle temporal gyrus (aMTG), posterior superior temporal gyrus (Wernicke’s area), anterior superior temporal gyrus (aSTG), and the temporal pole (temp pole) (Figure 1). The cognitive network was defined using the MDN atlas, the ToM atlas, and the DMN network from the CONN network parcellation atlas (Figure 1). These networks were included as a part of the cognitive network as the MDN, which is activated for tasks beyond language comprehension [47]; ToM is engaged during social reasoning and non-literal language comprehension [48]; and the DMN, which is critical in the construction of a narrative, i.e., in long-range temporal narratives [49].

The Harvard–Oxford atlas was also used to define ROIs for the perceptual and motor networks. The perceptual network included ROIs placed in the left hemisphere, specifically the occipital fusiform gyrus, the temporo-occipital fusiform cortex, the posterior temporal fusiform cortex, and the anterior temporal fusiform cortex (Figure 1). The visual word-form area (VWFA) is located within the left fusiform cortex [50]. It is known to support experience-driven responses and is associated with reading skills, as deficits in VWFA activity create a temporary failure in identifying letters and words [51,52,53]. The motor network included the left parietal operculum and precentral gyrus (Figure 1). The ventral sensorimotor cortex (also known as the secondary somatosensory cortex) is located within the left hemisphere of the parietal operculum [54]. It is involved in the production of speech sounds and the execution of speech articulation (i.e., speech motor plans) [55,56]. The finger, hand, and mouth areas of the sensorimotor cortex are located within the precentral gyrus [57,58]. These areas are recruited during the execution of motor movements during written and spoken speech [55,59]. The functional connectivity was averaged across all ROIs or atlases within the network of interest to create a single functional connectivity of that network. Within- and between-network connectivity was generated for each network. Within the language network, connectivity between ROIs was also extracted. Gray matter volumes for all regions within the language network and the total intracranial volume (TIV) were also calculated.

### 2.5. Statistical Analysis

Demographics and clinical characteristics were compared between the PCA and CU groups using Fisher’s exact test for categorical variables and Mann–Whitney test for continuous variables, as the data did not pass normality tests. Several normality tests (D’Agostino and Pearson, Anderson–Darling and Shapiro–Wilk tests) were performed to make an informed decision using the statistical software GraphPad Prism version 9.2.0.

For aim 1, the CONN functional connectivity toolbox [44] (www.nitrc.org/projects/conn, accessed on 13 August 2025) was used to generate maps for all networks within each group. Group average connectivity maps were corrected for false discovery rate (FDR) at *p* < 0.001 with cluster size correction for FDR at *p* < 0.05. The CONN toolbox was used to extract within- and between-network connectivity estimates. Multivariate linear regression models assessing within- and between-network connectivity in PCA compared to CU individuals were fit and adjusted for age and sex effects. Model 1 used the within-network connectivity of all networks as the outcome, which was predicted by diagnosis, while Model 2 used between-network functional connectivity between the language and all other networks as the outcome, which was predicted by diagnosis. Pairwise comparisons were performed after the models were fit. The variance inflation factor (VIF) was below 1.3 for all independent variables.

ROI-level maps were also generated for all ROIs within the language network for each group. Group average connectivity maps were corrected for FDR at *p* < 0.05 with cluster size correction for FDR at *p* < 0.05. Multivariate linear regression models assessing between-network connectivity from Broca’s area (inferior frontal gyrus) and Wernicke’s area (posterior superior temporal gyrus) across the other ROIs were fit and adjusted for age and sex effects. Separate models were fit for Broca’s and Wernicke’s areas, and these models used between-network connectivity as the outcome predicted by diagnosis. The variance inflation factor (VIF) was below 1.3 for all independent variables. Connectivity was evaluated from Broca’s area, as it is considered to be an important hub in speech production [61,62], and Wernicke’s area, as it is considered to be critically involved in language comprehension [63].

For aim 2, multivariate linear regression models assessing the relationship between network connectivity and clinical performance on language comprehension (BDAE repetition and PPT word-word) and production (BNT-SF, BDAE repetition, letter, and animal fluency) tests were fit within PCA patients. These models were adjusted for age and sex effects. Model 1 used language comprehension scores as an outcome, which was predicted by within-network connectivity of the language network and by between-network connectivity between the cognitive, perceptual, and language networks. Separate models were fit for within- and between-network connectivity. Model 2 used language production scores as an outcome, which were predicted by within-network connectivity of the language network and by between-network connectivity between the cognitive, motor, and language networks. Separate models were fit for within- and between-network connectivity. The variance inflation factor (VIF) was below 1.5 for all independent variables. Model 3 only examined the significant relationship between between-network connectivity within the language network across language comprehension and production tests. Separate models were fit for each connectivity finding, and these models used language tests as the outcome predicted by between-network connectivity. The variance inflation factor (VIF) was below 1.3 for all independent variables.

For aim 3, a multivariate linear regression model assessing the relationship between language within-network connectivity and gray matter volumes within the language network composite was also fit within PCA patients. This model adjusted for age, TIV, and sex effects. Here, the model used gray matter volumes as an outcome, which was predicted by within-network connectivity of the language network. The variance inflation factor (VIF) was below 2.5 for all independent variables. The gray matter volume composite of the language network included all the ROIs within the language network. IBM SPSS statistics v28 was used to perform all multivariate linear regression analyses.

## 3. Results

### 3.1. Patient Demographics

Table 1 shows the demographic and clinical characteristics of PCA patients and CU individuals. PCA and CU individuals showed differences in education but did not differ in sex or age at the time of scan. On clinical testing, PCA patients performed worse on the MoCA compared to the CU individuals.

### 3.2. Within- and Between-Network Connectivity (Aim 1)

#### 3.2.1. Network Level: Language Processing System

**Figure 2:**
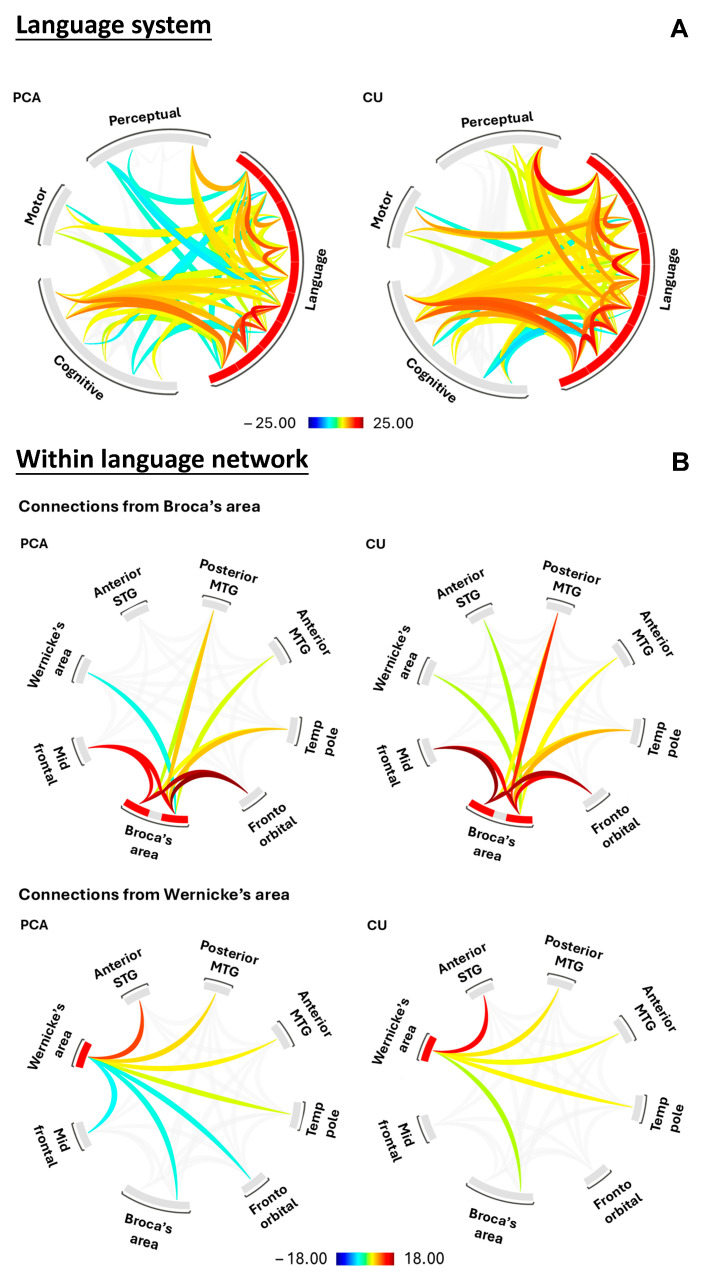
**Breakdown in functional connectivity within the language processing system.** These plots depict (**A**) the functional connectivity profiles in PCA and CU individuals. More specifically, they show a breakdown in communication to and from the language network. (**B**) The functional connectivity profiles in PCA and CU individuals. More specifically, they show a breakdown in communication at the regional level within the language network. Key: Broca’s area, inferior frontal gyrus, Wernicke’s area, posterior superior temporal gyrus.

Visual representations of the network-level maps for PCA and CU individuals are shown in Figure 2A. These maps draw attention to the stark difference in functional connectivity across both groups, with the PCA group showing reduced language within-network connectivity (fewer connections and lower connection strength) and between-network connectivity with other networks (fewer connections and lower connection strength) compared to CU individuals.

**Figure 3:**
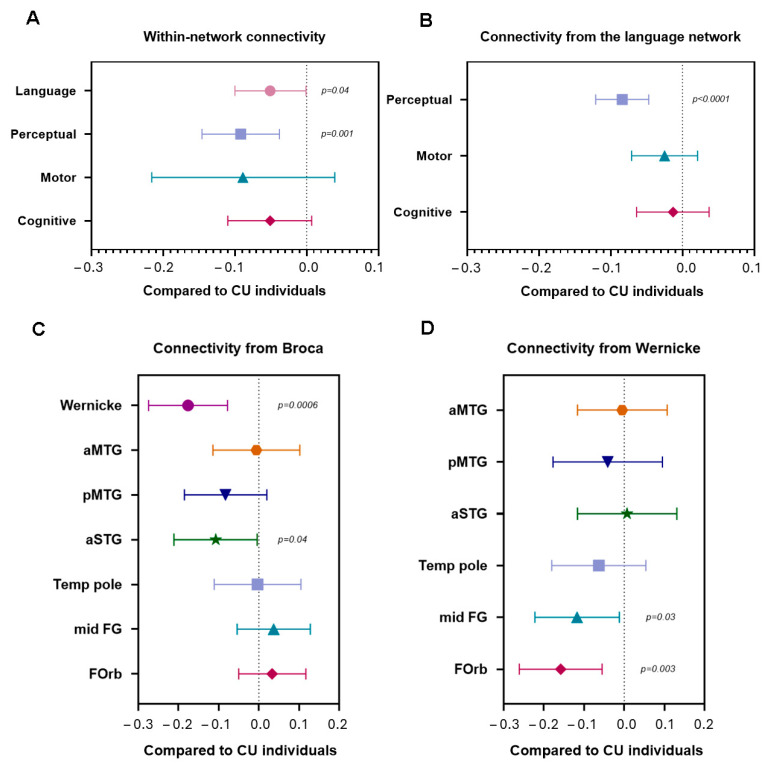
**Within-network connectivity and between-network connectivity within the language processing system.** These forest plots compare (**A**) within-network connectivity of all four networks in PCA compared to CU individuals, (**B**) between-network connectivity from the language network to other networks in PCA compared to CU individuals, (**C**) between-network connectivity from the Broca’s area to all other regions within the language system in PCA compared to CU individuals, and (**D**) between-network connectivity from the Wernicke’s area to all other regions within the language system in PCA compared to CU individuals. Plots show estimates and a 95% confidence interval. If the confidence interval does not touch zero (dashed line), the difference is considered significant. Key: Broca’s area, inferior frontal gyrus, Wernicke’s area, posterior superior temporal gyrus.

Multivariate linear regression models showed similar findings, with PCA showing reduced language within-network connectivity (*p* = 0.04) and perceptual within-network (*p* = 0.001) compared to CU individuals. No reductions were observed in the motor and cognitive networks (Figure 3A). The language and perceptual networks also showed reduced between-network connectivity (*p* < 0.0001) (Figure 3B).

#### 3.2.2. Regional Level: Within the Language Network

Visual representation of the regional level maps within the language network in PCA and CU individuals is shown in Figure 2B. These maps showed minor differences in functional connectivity (fewer connections and lower connection strength) in PCA when evaluating connectivity from Broca’s (inferior frontal gyrus) and Wernicke’s (posterior superior temporal gyrus) areas compared to CU individuals.

Multivariate linear regression models showed similar findings, with PCA showing reduced between-network connectivity with Broca’s area, specifically a decrease in Broca-Wernicke connectivity (*p* = 0.0006) and Broca-aSTG connectivity (*p* = 0.04) compared to CU individuals (Figure 3C). Reduced between-network connectivity with Wernicke’s area was also noted in PCA, with a decrease in Wernicke-midFG connectivity (*p* = 0.03) and Wernicke-FOrb connectivity (*p* = 0.003) compared to CU individuals (Figure 3D).

### 3.3. Relationship Between Clinical Scores and Language System Connectivity (Aim 2)

#### 3.3.1. Network Level: Language Processing System

PCA showed no association between language within-network connectivity and language comprehension scores from BDAE repetition and PPT word-word tests, as well as language production scores from BNT-SF, BDAE repetition, letter fluency, and animal fluency tests (Supplementary Table S1). Additionally, no associations were noted between language processing scores and between-network connectivity with the language network (Supplementary Table S2).

#### 3.3.2. Regional Level: Within the Language Network

**Figure 4:**
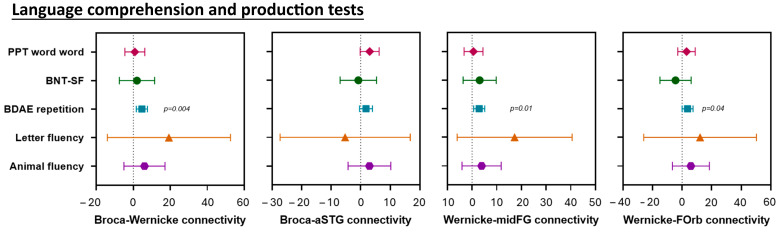
Relationship between regional connectivity within the language network and clinical performance on language processing tests. These forest plots compare clinical performance on language processing tests and Broca-Wernicke connectivity, Broca-aSTG connectivity, Wernicke-midFG connectivity and Wernicke-FOrb connectivity. Plots show estimates and 95% confidence interval. If the confidence interval does not touch zero, the difference is considered significant.

Within the language network, PCA showed positive associations between BDAE repetition scores and Broca-Wernicke (*p* = 0.004), Wernicke-midFG (*p* = 0.01), and Wernicke-FOrb (*p* = 0.04) connectivity. Hence, worse (lower) scores on the BDAE repetition were associated with lower connectivity (Figure 4).

### 3.4. Effect of Gray Matter Volumes on Language System Connectivity (Aim 3)

PCA did not show any associations between gray matter volume of the language network composite and language within-network connectivity (Supplementary Table S3).

## 4. Discussion

This study investigated the breakdown of the language processing system in PCA. Within the language processing system, we found reduced language within-network connectivity and perceptual within-network connectivity, along with reduced between-network connectivity between the language and perceptual networks in PCA. Breakdown in regional connectivity within the language network was also noted. These regional disruptions were associated with performance on the BDAE repetition test. Lastly, gray matter volume was not associated with connectivity in the language network.

### 4.1. Functional Connectivity Breakdown in the Language Processing System

Our previous studies showed a reduced within-network connectivity of the classical language network, which included only the core language hubs, i.e., Broca (inferior frontal gyrus) and Wernicke (posterior superior temporal gyrus) areas [6,14]. We extend these findings by showing reduced within-network connectivity in the extended language network, which, in addition to the classical language hubs, included several other brain areas that are specialized for language, including the inferior frontal orbital gyrus, middle frontal gyrus, posterior middle temporal gyrus, anterior middle temporal gyrus, anterior superior temporal gyrus, and the temporal pole. We further explored the interplay between these regions to better understand the breakdown of connectivity within the extended language network. We found reduced between-network connectivity between Broca’s and Wernicke’s areas, Broca’s area and the anterior superior temporal gyrus, Wernicke’s area and the middle frontal gyrus, and finally between Wernicke’s area and the inferior frontal orbital gyrus. We previously reported reduced connectivity between Broca’s and Wernicke’s areas in PCA [6,14]. Most PCA patients show greater atrophy and tau burden in the superior temporal gyrus when compared to CU individuals [13], so it would be logical to assume a disruption in functional connectivity may exist and stem from this brain region. Frontal brain regions such as midFG and FOrb are relatively spared in PCA [64]. Based on these findings, one can hypothesize an anterior–posterior communication disruption in these regions. Previous literature supports a similar anterior–posterior communication disruption between Broca’s area and the middle temporal gyrus in the language variant of AD and non-fluent and semantic variants of primary progressive aphasia [65,66]. Furthermore, the previous literature has shown disruptions in white matter tracts of the inferior fronto-occipital fasciculus (implicated in language cognition) in PCA [67]. Using [18F] fluorodeoxyglucose (FDG) PET, we have previously shown that PCA patients with more left-sided hypometabolism are more likely to develop aphasia [68].

The perceptual network also showed a significant reduction in within- and between-network connectivity with the language network. Since the perceptual network is in constant communication with the language network and actively involved in perceiving and delivering linguistic messages during language comprehension [19], one could theorize that any deficits in language comprehension identified in PCA may stem from a breakdown in the perceptual network or a breakdown in the communication between these two networks. However, no significant associations were noted with language comprehension tests in this study, which could suggest that the perceptual network may be affected for reasons completely unrelated to the language network. Interestingly, no breakdown was noted within the cognition network. We expected a breakdown within this network, as PCA patients show significant impairment in cognition [33] and breakdown in DMN connectivity [6,69]. One reason for the absence of a relationship could be that the cognition network accounted for connectivity in the MDN and ToM networks in addition to the DMN. So far, no studies have explored the functional connectivity networks of MDN and ToM in PCA, and it is not known to what extent these networks are affected in PCA. Furthermore, the previous literature also suggests the possibility of preserved inner speech in aphasia patients [70]. Hence, further studies exploring these associations are warranted. The motor network did not show any breakdown in the within- and between-network connectivity with the language network, which is reasonable, as language production skills are relatively preserved in PCA [9].

### 4.2. Relationship Between Clinical Scores, Gray Matter Volume and Language System Connectivity

As expected, no relationship was noted between the health of the language network and the clinical performance of language comprehension and production tests. Likewise, between-network connectivity across networks did not show any associations with clinical performance on language comprehension and production tests. These findings seem plausible, as language comprehension and production skills are typically spared in PCA [9]. In our PCA cohort, language comprehension skills were preserved, and production skills were relatively spared. However, when we focused on regional connectivity within the language network, worse performance on BDAE repetition was associated with a significant breakdown in Broca-Wernicke, Wernicke-midFG, and Wernicke-FOrb connectivity, with a trend (*p* = 0.10) for breakdown in Broca-aSTG connectivity. Deficits in repetition have been noted in PCA patients [9], so based on these findings, one could theorize that these deficits may stem from a breakdown in connectivity between the anterior and posterior language networks. This is in line with previous literature that reports a positive correlation between poor repetition and thinning of temporoparietal areas in the left hemisphere [71]. There is the possibility that these associations were driven by disease severity; therefore, we re-ran these models while adjusting for MoCA scores. Worse performance on BDAE repetition was still associated with a breakdown in Broca-Wernicke connectivity (*p* = 0.02), which implies that this finding results from language impairment. The associations between BDAE repetition and Wernicke-midFG and Wernicke-FOrb connectivity were no longer significant, suggesting that these findings may have been confounded by disease severity. The absence of a relationship between connectivity breakdown and clinical performance on BNT-SF and fluency tests was particularly thought-provoking. A decline in fluency has been noted previously in PCA patients [7], but we know that this decline is strongly mediated by left frontal brain regions [72,73], which are typically spared in PCA [64]; therefore, the absence of a relationship is understandable. Likewise, PCA patients also show deficits in confrontation naming (i.e., anomia) [9], which is measured by performance on the BNT-SF test [40]. However, BNT-SF testing relies heavily on visual processing, i.e., the ability to identify objects shown in pictures during the test administration, which is a major confounder for patients with PCA [13,15]. Moreover, in PCA, BNT scores have been correlated with left occipital volumes [12], indicating involvement of regions outside the language network. As expected, we did not see any associations with performance on the PPT word–word, as this test is most commonly used for assessing word knowledge [38], which is not affected in PCA [9]. Lastly, no relationship was noted between language within-network connectivity and volumes of the language network composite, which could suggest that these findings may not be confounded by volume.

### 4.3. Strengths, Limitations, and Future Directions

The strengths of this study include extensive clinical evaluations, consistent neuroimaging protocols across the PCA and CU groups, and models that control for differences in age and sex effects. Potential limitations of the study included the absence of more comprehensive speech-language testing and exploration of communication between the language processing system and other core networks of the disease. Future studies exploring these associations are warranted. Studies investigating associations with auditory comprehension and spontaneous speech are also needed.

## 5. Conclusions

Our findings highlight the disruptions that occur in the language processing system in PCA. PCA patients showed breakdown in the extended language network connectivity, along with regional disruptions within the language network. Although the perceptual network showed a breakdown, no associations were noted with language comprehension in PCA. At the network level, the language network did not influence clinical performance on language processing tests, but evaluating these relationships at the regional level did show a significant association with sentence repetition deficits. Together, these findings highlight the nature of language dysfunction in PCA and pave the way for employing language network connectivity as a disease biomarker in PCA.

## Figures and Tables

**Table 1:** Participants’ demographics and disease characteristics.

	PCA (*n* = 37)	CU (*n* = 39)	*p*-Value
Female, *n* (%)	22 (60%)	27 (69%)	0.47
Education, yr	16 (13, 18)	18 (16, 18)	**0.07**
Age at onset, yr	58.2 (54.9, 61.1)	-	-
Age at scan, yr	62.7 (59.5, 66.2)	61.6 (56.4, 66)	0.17
Time from onset to scan, yr	4.28 (3.07, 5.86)	-	-
MoCA (30)	17 (10, 20)	27 (26, 28)	**<0.0001**
Simultanagnosia (20)	7 (3, 11)	-	-
VOSP Cube Analysis (10)	1 (0, 3)	-	-
VOSP Incomplete Letters (20)	11 (6, 15)	-	-
BNT-SF (15)	12 (9.8, 14)	-	-
BDAE repetition (10)	9 (8, 10)	-	-
Letter fluency	32 (23, 38.5)	-	-
Animal fluency	13.5 (8.5, 15)	-	-
PPT word-word (52)	50 (49, 51)	-	-

Data shown are *n* (%) or median (first and third quartiles). For continuous variables, *p*-values are from Mann–Whitney test. For categorical variables, *p*-values are from Fisher’s exact test. Significant p values are highlighted in bold text. Key: CU, cognitively unimpaired; PCA, Posterior Cortical Atrophy; MoCA, Montreal Cognitive Assessment Battery; VOSP, Visual Object and Space Perception Battery; PPT, Pyramids and Palm Trees; BNT-SF, Boston Naming Test- Short Form; BDAE, Boston Diagnostic Aphasia Exam.

## Data Availability

The data that support the findings of this study will be available from the corresponding author on request, as we have an internal review protocol in place for data requests. All data will be made publicly available by the end of the grant.

## Supplementary Materials

The following supporting information can be downloaded at: https://www.mdpi.com/article/10.3390/brainsci15121287/s1, Table S1. The validity and reliability scores for clinical tests; Table S2. Linear regression results investigating the relationship between functional connectivity and clinical performance; Table S3. Linear regression results investigating the relationship between functional connectivity and gray matter volume. References [74,75,76,77,78,79,80,81] were cited in Supplementary Materials.

## Abbreviations

The following abbreviations are used in this manuscript:

AD	Alzheimer’s Disease
CU	Cognitively Unimpaired individuals
MoCA	Montreal Cognitive Assessment
MRI	Magnetic Resonance Imaging
MPRAGE	Magnetization-prepared rapid gradient echo
NRG	Neurodegenerative Research Group
PiB	Pittsburgh Compound B
PCA	Posterior Cortical Atrophy
PET	Positron Emission Tomography
ROI	Regions of Interest
rsfMRI	Resting state functional MRI
BNT-SF	15-item Boston Naming Test- Short form
BDAE	Boston Diagnostic Aphasia Exam
PPT	Pyramids and Palm Trees
VOSP	Visual Object and Space Perception Battery
