# Supplementary material for: Posterior Cortical Atrophy: Altered Language Processing System Connectivity and Its Implications on Language Comprehension and Production
Source: Brain Sci. 2025 Nov 29;15(12):1287. doi: 10.3390/brainsci15121287 (PMC12730223; doi:10.3390/brainsci15121287)
Supplement: Supplementary file 1 [file brainsci-15-01287-s001.zip › brainsci-3988439-supplementary.pdf]

Table S1. The validity and reliability scores for clinical tests

Clinical test	Validity scores	Reliability scores	Reference
MoCA	Sensitivity = 100%	Correlation, r = 0.92	1
	Specificity = 87%		
VOSP cubes	Sensitivity = 75%	-	2
	Specificity = 68%		
VOSP incomplete letters	Sensitivity = 88%	-	2
	Specificity = 42%		
PPT word-word	Sensitivity = 63%	Correlation, r = 0.47	3 and 4
	Specificity = 85%		
BNT-SF	Sensitivity = 38%	Cronbach's alpha =	5 and 6
	Specificity = 96%	0.72 – 0.86.	
Letter fluency	Sensitivity = 89%	Correlation, r = 0.74	7 and 8
	Specificity = 85%		
Animal fluency	Sensitivity = 100%	Correlation, r = 0.71	7 and 9
	Specificity = 93%		

Key: MoCA, Montreal Cognitive Assessment Battery; VOSP, Visual Object and Space Perception Battery; PPT, Pyramids and Palm Trees; BNT-SF, Boston Naming Test- Short Form

References:

1. Nasreddine, Z. S., Phillips, N. A., Bédirian, V., Charbonneau, S., Whitehead, V., Collin, I., Cummings, J. L., & Chertkow, H. (2005). *The Montreal Cognitive Assessment, MoCA: A brief screening tool for mild cognitive impairment.* **Journal of the American Geriatrics Society**, **53**(4), 695–699.
2. Quental, N. B. M., Brucki, S. M. D., & Bueno, O. F. A. (2013). *Visuospatial Function in Early Alzheimer's Disease — The Use of the Visual Object and Space Perception (VOSP) Battery*. *PLOS ONE*, 8(7), e68398.
3. Stockbridge, M. D., Tippett, D. C., Breining, B. L., Vitti, E., & Hillis, A. E. (2021). *Task performance to discriminate among variants of primary progressive aphasia*. *Cortex*, 145, 201–211.

4. Klein, L. A., & Buchanan, J. A. (2009). *Psychometric properties of the Pyramids and Palm Trees Test. Journal of Clinical and Experimental Neuropsychology, 31*(7), 803–808.
5. Abeare, K. B., Cutler, L., An, K. Y., Razvi, P., Holcomb, M., & Erdodi, L. A. (2022). *BNT-15: Revised performance validity cutoffs and proposed clinical classification ranges. Cognitive and Behavioral Neurology, 35*(3), 155–168.
6. Attridge, J., Zimmerman, D., Rolin, S., & Davis, J. (2022). *Comparing Boston Naming Test short forms in a rehabilitation sample. Applied Neuropsychology: Adult, 29*(4), 810–815.
7. Monsch, A. U., Bondi, M. W., Butters, N., Salmon, D. P., Katzman, R., & Thal, L. J. (1992). *Comparisons of verbal fluency tasks in the detection of dementia of the Alzheimer type. Archives of Neurology, 49*(12), 1253–1258.
8. Tombaugh, T. N., Kozak, J., & Rees, L. (1999). *Normative data stratified by age and education for two measures of verbal fluency: FAS and animal naming. Archives of Clinical Neuropsychology, 14*(2), 167–177.
9. St-Hilaire, A., Hudon, C., Vallet, G. T., Bherer, L., Lussier, M., & Macoir, J. (2016). *Normative data for phonemic and semantic verbal fluency test in the adult French-Quebec population and validation study in Alzheimer's disease and depression. The Clinical Neuropsychologist, 30*(7), 1126–1150.

Table S2. Linear regression results investigating the relationship between functional connectivity and clinical performance

Clinical tests	Functional connectivity	Estimate	Lower bound	Upper bound	p value
**Language comprehension**					
BDAE repetition	Within language network	3.30	-2.98	9.59	0.28
	Language-perceptual	2.64	-6.33	11.61	0.55
	Language-cognitive	-0.81	-7.31	5.68	0.79
PPT word-word	Within language network	0.51	-8.46	9.48	0.90
	Language-perceptual	7.55	-4.47	19.59	0.20
	Language-cognitive	3.85	-4.86	12.56	0.37
**Language production**					
BNT-SF	Within language network	-6.71	-20.89	7.47	0.33
	Language-motor	3.79	-15.46	23.05	0.68
	Language-cognitive	-14.99	-30.73	0.74	0.06
BDAE repetition	Within language network	3.75	-1.65	9.16	0.16

	Language-motor	-1.16	-8.85	6.51	0.75
	Language-cognitive	0.44	-5.83	6.72	0.88
Letter fluency	Within language network	-21.6	-80.84	37.45	0.45
	Language-motor	-1.79	-85.18	81.58	0.96
	Language-cognitive	-36.90	-105.03	31.21	0.27
Animal fluency	Within language network	-8.87	-25.77	8.02	0.28
	Language-motor	-13.75	-37.62	10.12	0.24
	Language-cognitive	1.446	-18.061	20.953	0.880

Multivariate linear regression models adjusting for age and sex effects were fit to assess the relationship between network connectivity and clinical performance on language comprehension (BDAE repetition and PPT word-word) and production (BNT-SF, BDAE repetition, letter, and animal fluency) tests within PCA patients.

Table S3. Linear regression results investigating the relationship between functional connectivity and gray matter volume

Gray matter volume	Functional connectivity	Estimate	Lower bound	Upper bound	p value
Language network composite	Within-network connectivity of the language network	7862.41	-3405.74	19130.57	0.16

Multivariate linear regression models adjusting for age, TIV, and sex effects were fit to assess the relationship between within-network connectivity of the language network and gray matter volumes of the language network composite within PCA patients.
